# Editorial: Emerging SARS-CoV-2 variants: Genomic variations, transmission, pathogenesis, clinical impact and interventions

**DOI:** 10.3389/fmed.2023.1178696

**Published:** 2023-03-29

**Authors:** Pragya D. Yadav, Sanjay Kumar, Éric Bergeron, Meerjady Sabrina Flora

**Affiliations:** ^1^Indian Council of Medical Research-National Institute of Virology, Pune, Maharashtra, India; ^2^Department of Neurosurgery, Command Hospital (Southern Command), Armed Forces Medical College (AFMC), Pune, India; ^3^Centers for Disease Control and Prevention (CDC), Atlanta, GA, United States; ^4^Directorate General of Health Services, Dhaka, Bangladesh

**Keywords:** genomic variations, transmission, pathogenesis, clinical impact, intervention

Severe acute respiratory syndrome coronavirus-2 (SARS-CoV-2) originated in Wuhan in December 2019 and rapidly spread across the globe, with the World Health Organization (WHO) declaring it a pandemic in March 2020. Since early 2021, multiple SARS-CoV-2 variants have emerged in various countries around the world (1). Among these, the “Variant of Concern” has been reported to be highly transmissible, infectious, and capable of evading the natural or vaccine-induced immune response. The rapid spread of these variants has resulted in a daily increase in SARS-CoV-2 cases, which are associated with severe morbidity and mortality, exacerbating the pandemic situation (2). The newly emerged variants have become a serious threat to the global COVID-19 vaccination program due to their reduced susceptibility to currently available vaccines (3). It is critical to conduct active genomic surveillance of SARS-CoV-2 variants in order to better understand their transmission, pathogenesis, and the efficacy of vaccines and other therapeutics against these variants.

This Research Topic was created to explore the global emergence and prevalence of SARS-CoV-2 variants, key mutations impacting transmission, infectivity, pathogenicity, and associated immune escape against vaccines and other therapeutics. The 64 papers that comprise this Research Topic, accepted by authors of various countries and continents, investigate mainly genomic variations of SARS-CoV-2, the clinical impact of COVID-19, and the interventions to control the pandemic.

The mutational landscape on the SARS-CoV-2 structural and non-structural proteins and their impact on diagnostics, therapeutics, and vaccines have been studied across different geographical locations worldwide. Many of the authors tried to determine the main characteristics of the emerging various variants of SARS-CoV-2, including their distribution, mutations, transmissibility, severity, and susceptibility to immune responses. All these studies revealed different mutational patterns and their impact on diagnostics, their role in immune evasion, pathogenesis, and advanced research on current vaccines and therapeutics, i.e., the emergence of Variants of Concern and Variants of Interest, novel mutations, rare double-deletion variation in the spike region (68–76del+spike 675–679del), dynamism in intrahost single nucleotide variants (iSNVs) in the severe and mild infection cohorts, immune evasion of the Mu variant and a derivative of the Delta strain with E484K and N501Y mutations, increased virulence and transmission, and the functional role of ORF8 in viral pathogenesis (Agius et al.; Biswas et al.; Bittar et al.; Cheng et al.; Deval et al.; Ha et al.; Huang et al.; Jiang et al.; Khan et al.; Levi et al.; Li T. et al.; Liu C. et al.; Liu L.-T. et al.; Miyakawa et al.; Mostefai et al.; Negrón et al.; Osman et al.; Qin et al.; Smith et al.; Takatsuka et al.; Thakur et al.; Zhang, Ejikemeuwa et al.; Zhang, Hu et al.; Zimerman et al.). In addition, these many studies also reported shedding of SARS-CoV-2, nosocomial transmission in a cluster of children with underlying malignancy, isolation of SARS-CoV-2 variants and assessment of their genetic diversity, cell tropism and interaction with proteins that promote virus entry, and animal pathogenesis with SARS-CoV-2 variants (Ding et al.; Fernandez et al.; Lavania et al.; Madi et al.; Mostefai et al.; Praharaj et al.; Putri et al.; Rodriguez-Sevilla et al.; Singh et al.; Zhang Y.-D. et al.). These findings will be of value to the development of next-generation vaccines and therapeutic antibodies.

Ending the COVID-19 pandemic necessitates a shared understanding of the SARS-CoV-2 and COVID-19 mechanisms. The studies demonstrated the weighted network modeling of frequency trajectories of mutations (FTMs), the Global Evaluation of SARS-CoV-2/hCoV-19 Sequences 2 (GESS v2), and how IdbSV and logistic regression models enable us to rapidly and easily track down SARS-CoV-2 variants, identify SNVs, and spike binding parameters of ACE2 (Essabbar et al.; Huang et al.; Li K. et al.).

Various studies have also reported the usefulness of real-time RT-PCRs for the detection of the relevant mutations/deletions present in the Spike protein in VOC/VOIs, amplicon-based genome sequencing with MinION, utilizing the ARTIC V4 primers for improving genome recovery of SARS-CoV-2 variants, and HiSpike, a method for high-throughput cost-effective targeted next-generation sequencing of the spike gene. These studies highlight the importance of whole genome sequencing and expanded real-time monitoring of diagnostic PCR assays during a pandemic (Angulo et al.; Castro et al.; Fass et al.; Lambisia et al.; Park et al.; Rafiqul Islam et al.).

The COVID-19 pandemic has been a great challenge to public health systems. The factors associated with a higher risk of death were those related to coinfection and comorbidities (Alfonso-Sanchez et al.; Angulo et al.; Hosch et al.; Pang et al.). Although the emergence of vaccines may reduce the threat posed by SARS-CoV-2 variants, these variants are still very important and need to be studied. Many of the studies focused on the various aspects of COVID-19, mainly a wide range of clinical presentations, viral transmissibility, viral load, disease severity, lethality, breakthrough infections, reinfections, waning response with natural or vaccine-induced immunity, and the need for booster vaccination (Colavita et al.; da Silva et al.; Guo et al.; Hu et al.; Isnaini et al.; Koyama et al.; Li R. et al.; Xu et al.; Shastri et al.; Temsah et al.; Thangaraj et al.). Moreira-Soto et al. also demonstrated that the equine polyclonal antibodies (pAbs) efficiently neutralize the variants of concern alpha, beta, epsilon, gamma, and delta.

Few studies demonstrated the usefulness of fluorescently labeled lateral flow assays enabling rapid detection of SARS-CoV-2 under field or at-home conditions and in-house IgG ELISA and PRNT in vaccine efficacy studies (Auerswald et al.; Gesto et al.; Walish et al.). Some research findings also revealed the ineffectiveness of red blood cell distribution width (RDW) as a prognostic factor for COVID-19 severity, potential adverse perinatal outcomes with the Gamma variant, and the importance of a booster vaccination in patients with rheumatoid arthritis (Fernandez et al.; Zhang, Ejikemeuwa et al.; Zhao et al.).

The outcome of SARS-CoV-2 infection varies from asymptomatic, symptomatic to fatal cases. This data highlights the importance of better-adapted non-pharmacological measures and clinical discharge of patients in order to prevent the spread of SARS-CoV-2 to the general population (Cunha et al.). Currently available COVID-19 vaccines are still effective against the SARS-CoV-2 variant. If vaccine effectiveness against SARS-CoV-2 variants continues to deteriorate, the pandemic could worsen. To address the new challenges posed by new SARS-CoV-2 variants, multiple measures should be implemented, including public health interventions, vaccination expansion, and the development of a new vaccine booster.

All these research findings emphasized the importance of monitoring vaccine breakthrough infections, through the characterization of virological, immunological, and clinical features associated with these events, in order to tune prevention measures in the next phase of the COVID-19 pandemic.

## Author contributions

PY wrote the first draft. All authors reviewed and approved the final manuscript.
