# Corrigendum: Editorial: Emerging SARS-CoV-2 variants: genomic variations, transmission, pathogenesis, clinical impact and interventions

**DOI:** 10.3389/fmed.2023.1260601

**Published:** 2023-07-27

**Authors:** Pragya D. Yadav, Sanjay Kumar, Éric Bergeron, Meerjady Sabrina Flora

**Affiliations:** ^1^Indian Council of Medical Research-National Institute of Virology, Pune, Maharashtra, India; ^2^Department of Neurosurgery, Command Hospital (Southern Command), Armed Forces Medical College (AFMC), Pune, India; ^3^Centers for Disease Control and Prevention (CDC), Atlanta, GA, United States; ^4^Directorate General of Health Services, Dhaka, Bangladesh

**Keywords:** genomic variations, transmission, pathogenesis, clinical impact, intervention

In the published article, there was an error. “ORF8” was incorrectly written as “ORF8a”.

A correction has been made to Paragraph Number 3. This sentence previously stated: “All these studies revealed different mutational patterns and their impact on diagnostics, their role in immune evasion, pathogenesis, and advanced research on current vaccines and therapeutics, i.e., the emergence of Variants of Concern and Variants of Interest, novel mutations, rare double-deletion variation in the spike region (68-76del+spike 675–679del), dynamism in intrahost single nucleotide variants (iSNVs) in the severe and mild infection cohorts, immune evasion of the Mu variant and a derivative of the Delta strain with E484K and N501Y mutations, increased virulence and transmission, and the functional role of **ORF8a** in viral pathogenesis.”

The corrected sentence appears below: “All these studies revealed different mutational patterns and their impact on diagnostics, their role in immune evasion, pathogenesis, and advanced research on current vaccines and therapeutics, i.e., the emergence of Variants of Concern and Variants of Interest, novel mutations, rare double-deletion variation in the spike region (68–76del+spike 675–679del), dynamism in intrahost single nucleotide variants (iSNVs) in the severe and mild infection cohorts, immune evasion of the Mu variant and a derivative of the Delta strain with E484K and N501Y mutations, increased virulence and transmission, and the functional role of **ORF8** in viral pathogenesis.”

The authors apologize for this error and state that this does not change the scientific conclusions of the article in any way. The original article has been updated.

